# Being a woman in sports arenas: the relationship between gender role stress and sports motivation

**DOI:** 10.3389/fpubh.2025.1734463

**Published:** 2026-01-08

**Authors:** Ilimdar Yalcin, Burcu Sıla Sezer, Yakup Kilic, Laurentiu-Gabriel Talaghir, Cristina-Corina Bentea, Dumitru Marius Cosoreanu

**Affiliations:** 1 Faculty of Sport Sciences, Bingol University, Bingol, Türkiye; 2 Faculty of Sport Sciences, Firat University, Elazig, Türkiye; 3 Faculty of Physical Education and Sport, Dunarea de Jos University of Galati, Galati, Romania

**Keywords:** female athletes, gender role stress, sport motivation, sport participation, women in sports

## Abstract

**Introduction:**

Gender roles continue to shape women’s participation and experiences in sport, often imposing psychological pressures that influence their performance and motivation. Understanding how gender role-related stress affects female athletes’ motivation is essential for promoting equitable and supportive sport environments. The aim of this study was to examine the extent to which female athletes’ perceptions of gender role stress influence their sport motivation.

**Methods:**

This study employed a relational survey design. The study group consisted of 138 female participants studying at a faculty of sport sciences and engaged in various sports branches. Data were collected using a personal information form, the Female Gender Role Stress Scale, and the Sport Motivation Scale. The data obtained in the study were analyzed using the SPSS package program. Normality analyses revealed that the data exhibited a normal distribution. Descriptive statistics, Pearson correlation analysis, and simple regression analyses were employed in the analysis process.

**Results:**

The regression analysis results revealed that female gender role stress had a low but significant associated effect on motivational regulation dimensions (identified regulation, integrated regulation, introjected regulation, and intrinsic motivation). In addition, the variable of years of sports experience was found to be significantly associated with identified, integrated, and intrinsic motivation.

**Conclusion:**

A positive and significant relationship was observed between gender role-related stress among female athletes stress and the dimensions of sport motivation. These results may be related to the fact that women exhibit slightly higher motivation for sports when they experience stress due to their gender roles.

## 1 Introduction

Human beings are social entities who continuously display behaviors within society. The factors influencing these behaviors have long been a subject of interest. Among these factors, gender plays a crucial role in understanding the underlying causes of behavior. Although often considered a biological concept (1), gender in fact represents a cultural construct encompassing the personality traits, expected roles, and responsibilities attributed to men and women (2–6). The most critical dimension of this cultural construct is rooted in deeply held beliefs and values (7, 8).

People differ in their gender beliefs. Some adopt traditional gender roles such as the “feminine woman” or the “masculine man,” whereas others may internalize both masculine and feminine roles to varying degrees (9). Supporting this notion, Beauvoir’s famous statement “One is not born, but rather becomes, a woman” emphasizes that gender differentiation is not only biological but also a psychological and cultural process (10, 11). Accordingly, the cultural values, beliefs, and attitudes of societies teach individuals how to behave as men or women, either in accordance with or contrary to their biological identities (12). This highlights the concept of gender, which refers to the relational nature of being male or female and the socially constructed roles associated with each (13–16). These socially constructed roles are considered one of the most significant factors distinguishing humans from animals (17). However, these roles, rather than being equally distributed, have historically been institutionalized in favor of men (18). As a result, gender-based inequalities have predominantly been applied to women (19), leading to various forms of deprivation of rights (20).

Historically, patriarchal cultural structures have reinforced male dominance (21). Men have often been associated with superior rationality, and in some belief systems even identified with the divine (22, 23). This inequality, evident in all aspects of society, becomes particularly pronounced in the field of sports (14). Sports environments are areas where individuals are frequently exposed to pressure, competition, and performance expectations (24). Although such environments may not explicitly distinguish between male and female participants, they are socially classified into masculine and feminine domains (25, 26). Many sports are therefore perceived as masculine domains due to their association with physical strength, competitiveness, and achievement (27). This division of sports along gender lines directly affects patterns of participation, specialization, and experiences in sport. Female athletes, in particular, often encounter greater discrimination and exclusion when participating in sports culturally defined as “masculine” (28, 29). Studies have shown that women who act against gender norms, especially in male-dominated sports, experience heightened levels of stress and pressure, which may negatively impact their motivation (30–32). Furthermore, due to the predominance of feminine sports compared to masculine ones, women’s participation in sports is generally less encouraged (33, 34). Female athletes must therefore not only maintain their athletic performance but also cope with societal expectations. Indeed, the balance female athletes strive to maintain socially and athletically can significantly impact their motivation and athletic stress levels (35, 36). Therefore, the psychological and social roles of female athletes should be examined within the context of sports. In support, Çam (37) stated that providing a positive psychological environment is essential for good performance. The psychological burden imposed by these societal expectations manifests as gender role stress (38). This stress can be defined as a type of social anxiety arising from the perception of failing to conform to gender norms or feeling inadequate in relation to them (39). Importantly, experiencing stress does not necessarily imply negative outcomes for individuals. Burton (40) emphasized that adequate and balanced levels of stress are associated with higher performance. In other words, improving performance requires achieving an optimal level of stress. Within the sports arena, athletes are continually confronted with both internal and external stressors (41). Motivation serves as the driving force regulating both internal and external factors (42, 43). Motivation is therefore a critical determinant in understanding the reasons behind behaviors (44). Derived from the root word motive (45), motivation develops in proportion to the satisfaction gained from the activity being undertaken (46). In sports settings, motivation determines not only participation but also continuity, making it a key factor in athletic success (47). Thus, both optimal stress and motivation play critical roles in sustaining participation in sports, overcoming challenges, and achieving success in terms of performance.

Indeed, a review of the literature revealed that the relationship between gender and motivation has not been specifically examined in the sample of female athletes (48). Studies on gender roles have generally been descriptive, and a lack of empirical studies in this regard has been noted. Furthermore, it has been determined that motivational assessments are limited, and detailed regulatory dimensions in this context have not been examined. Therefore, this gap in the literature has become even more pronounced in the cultural context. This study aims to contribute to a better understanding of research and practice in the field by offering insights into how female athletes manage gender role stress and how their athletic motivation can be directed accordingly.

## 2 Methodology

### 2.1 Research model

This In order to facilitate the interpretation of the collected data, the survey method was employed (49). Within this framework, a relational survey model was used in the study sample. The relational survey model is applied to determine the presence and degree of relationships between two or more variables and their associations. From this perspective, the study is of quantitative nature (50).

### 2.2 Participants

The study group consisted of 138 female students (age = 21.34 ± 2.48; years of doing sports = 4.04 ± 3.37) selected by convenience sampling method who were studying at Bingöl University Faculty of Sports Sciences and actively involved in various sports branches (taekwondo, archery, basketball, swimming, volleyball, boxing, handball, wrestling, football, athletics, bocce and tennis).

### 2.3 Data collection tools

All participants took part in the study voluntarily, were informed beforehand about its purpose, and provided consent in accordance with ethical standards. Data were gathered using a Personal Information Form created by the researcher to identify participants’ demographic characteristics, along with the Female Gender Role Stress Scale and the Sport Motivation Scale. The data collection process was carried out through face-to-face surveys, and each participant completed the questionnaire in approximately 6–8 min.

#### 2.3.1 Personal information form

To obtain descriptive information about the participants, researchers administered a form including questions regarding gender, age, years of sports experience, type of sport, and sport discipline.

#### 2.3.2 Female gender role stress scale

The Female Gender Role Stress Scale was developed by Koç et al. (51). The scale consists of 20 items and has a unidimensional structure (e.g., The notion that ‘a woman does not laugh out loud’; The perception that ‘cooking is a woman’s job’). It is scored on a 5-point Likert scale ranging from 1 (“Not at all stressful”) to 5 (“Extremely stressful”), and it does not include reverse-coded items. The minimum score possible is 20, and the maximum is 100, with higher scores indicating greater experiences of gender role stress. The Cronbach’s alpha value of the original scale was reported as 0.92 (51). In the present study, Cronbach’s alpha was found to be 0.97.

#### 2.3.3 Sport motivation scale-6

To measure athletes’ sports motivation, the Motivation in Sports Scale, developed by Mallet and colleagues (52) and adapted to Turkish by Demir (53), was used. The Motivation in Sports Scale has a six-factor structure (Amotivation, Identified Regulation, External Regulation, Integrated Regulation, Introjected Regulation and Intrinsic Motivation) and consists of 24 items (e.g., I have to do sports to feel good.). Items are answered on a 5-point Likert-type scale ranging from 1 (Strongly disagree) to 5 (Strongly agree) and does not include reverse-scored items. Higher scores indicate that athletes exhibit higher sports motivation. In the Turkish adaptation study, Cronbach’s *α* reliability coefficient was reported as 0.70. When Cronbach alpha values were examined in the study, the Amotivation dimension was found to be 0.77, the Identified Regulation dimension was 0.77, the External Regulation dimension was 0.68, the Integrated Regulation dimension was 0.83, the Introjected Regulation dimension was 0.82 and the Intrinsic Motivation dimension was 0.76.

### 2.4 Ethical approval

This research was ethically approved by the Bingol University Health Sciences Scientific Research and Publication Ethics Committee with decision number 6 taken at the meeting number 24/10 dated 07.05.2024.

### 2.5 Statistical analysis

Before starting the analysis, outliers were examined, and since no extreme outliers were identified, all data were included. To assess normality, skewness and kurtosis values were examined, and all scale dimensions were found to be within the ±1.5 reference range. Consistent with this, Tabachnick et al. (54) emphasized that values within this range indicate normal distribution. Spearman rank correlations and 1,000-sample bootstrap confidence intervals were calculated, along with normality assessments. The results were consistent with the correlation and regression patterns. Based on the findings, it was determined that the analyses did not deviate from normality and the results were consistent. Pearson correlation analysis was used to examine the relationships between years of sports participation and scale subdimensions, as well as among the subdimensions themselves. Simple linear regression analysis was performed to evaluate the predictive effects. A significance level of *p* < 0.05 was adopted for all analyses, which were conducted using the SPSS statistical software package.

## 3 Results

In this section, the statistical analyses conducted to examine the relationships between female gender role stress and sport motivation are presented. Prior to the main analyses, the data were tested for normality, and all variables were found to meet the assumptions required for parametric testing. Descriptive statistics, Pearson correlation coefficients, and simple linear regression analyses were subsequently employed to determine the nature and strength of the associations among variables. The results are organized to first present the descriptive characteristics of the participants, followed by the correlations between years of sport experience, gender role stress, and the dimensions of sport motivation. Finally, regression analyses are reported to assess the predictive power of female gender role stress on motivational regulation dimensions.

In Table 1, the skewness and kurtosis values of the scale dimensions used in the study were examined, and it was found that the skewness values ranged between −0.941 and 0.019, while the kurtosis values ranged between −0.464 and 0.143.

**Table 1:** The examination of the mean, standard deviation, skewness, and kurtosis values of the female gender role stress scale (FGRSS) and the sport motivation scale (SMS-6).

Scales	Variables	n	Mean	SD	Skewness	Kurtosis
FGRSS	Total score	138	3.73	1.13	−0.941	0.083
SMS-6	Amotivation	138	11.16	3.77	−0.022	−0.451
	Identified regulation	138	14.63	3.29	−0.329	0.114
	External regulation	138	12.64	3.48	0.019	−0.464
	Integrated regulation	138	14.60	3.65	−0.372	−0.434
	Introjected regulation	138	14.80	3.47	−0.612	0.143
	Intrinsic motivation	138	14.50	3.35	−0.412	0.049

According to the results of the multiple correlation analysis, no statistically significant relationship was found between years of sports experience participation and the female gender role stress dimension (n(138) = −0.092; *p* > 0.05). However, years of sports experience showed a positive and low-level correlation with the identified regulation dimension (n(138) = 0.234, *p* < 0.01), the integrated regulation dimension (n(138) = 0.282**; *p* < 0.01), and the intrinsic motivation dimension (n(138) = 0.223**; *p* < 0.01) of sport motivation (Table 2).

**Table 2:** Pearson correlations between the female gender role stress scale and the sport motivation scale dimensions by years of sports experience.

Scales	Dimensions	Years of sports experience	
FGRSS	Total score	*r*	−0.092
		*p*	0.281
SMS-6	Amotivation	*r*	−0.081
		*p*	0.346
	Identified regulation	*r*	0.234**
		*p*	0.006
	External regulation	*r*	0.050
		*p*	0.557
	Integrated regulation	*r*	0.282**
		*p*	0.001
	Introjected regulation	*r*	0.130
		*p*	0.129
	Intrinsic motivation	*r*	0.223**
		*p*	0.009

*p <* 0.01**.

According to the results of the multiple correlation analysis, a positive and low-level significant relationship was found between the female gender role stress dimension and the sport motivation dimensions of identified regulation (*r* = 0.191; *p* < 0.05), integrated regulation (*r* = 0.174; *p* < 0.05), introjected regulation (*r* = 0.204*; *p* < 0.05), and intrinsic motivation (*r* = 0.171*; *p* < 0.05; Tables 2, 3).

**Table 3:** Pearson Correlation analysis results for the relationship between the female gender role stress scale (FGRSS) and the sport motivation scale (SMS-6) dimensions.

Scales	Dimensions		1	2	3	4	5	6	7
FGRSS	Total Score (1)	*r*	1						
		*p*							
SMS-6	Amotivation (2)	*r*	0.075	1					
		*p*	0.385						
	Identified Regulation (3)	*r*	0.191*	0.001	1				
		*p*	0.025	0.996					
	External Regulation (4)	*r*	0.164	0.258**	0.611**	1			
		*p*	0.055	0.002	0.001				
	Integrated Regulation (5)	*r*	0.174*	−0.135	0.830**	0.535**	1		
		*p*	0.041	0.115	0.001	0.001			
	Introjected Regulation (6)	*r*	0.204*	−0.025	0.817**	0.476**	0.772**	1	
		*p*	0.016	0.768	0.001	0.001	0.001		
	Intrinsic Motivation (7)	*r*	0.171*	−0.087	0.804**	0.487**	0.780**	0.725**	1
		*p*	0.045	0.308	0.001	0.001	0.001	0.001	

*p <* 0.05*; *p* < 0.01**.

According to the results of the simple linear regression analysis presented in Table 4, female gender role stress was found to have a significant effect on the identified regulation dimension, accounting for 3.6% of the variance (R^2^ = 0.036, *p* < 0.05). Female gender role stress was found to have a significant effect on the integrated regulation dimension, accounting for 3% of the variance (R^2^ = 0.030; *p* < 0.05). Female gender role stress was found to have a significant effect on the introjected regulation dimension, accounting for 4.2% of the variance (R^2^ = 0.042; *p* < 0.05). Female gender role stress was found to have a significant effect on the intrinsic motivation dimension, accounting for 3% of the variance (R^2^ = 0.029; *p* < 0.05). When the analysis results are examined, it is noted that the findings are significant but the effect level is low. Additionally, when the semi-partial effect size (sr^2^ = 0.037) and Cohen’s f^2^ value (f^2^ = 0.036) were examined, the dependent variable, Identified Regulation (sr^2^ = 0.037; f^2^ = 0.036), Integrated Regulation, (sr^2^ = 0.031; f^2^ = 0.030), Introjected Regulation (sr^2^ = 0.044; f^2^ = 0.041) and Intrinsic Motivation (sr^2^ = 0.030; f^2^ = 0.029) was found to be low.

**Table 4:** Regression analysis results predicting the dependent variable.

Dependent Variable	Variables	β	t	*p*	R^2^	_Adj_R^2^	F	f^2^	sr^2^
Identified regulation	Constant		13.213	0.000	0.036	0.029	5.122	0.037	0.036
	FGRSS Total	0.191	2.263	0.025					
Integrated regulation	Constant		11.849	0.000	0.030	0.023	4.237	0.031	0.030
	FGRSS Total	0.174	2.058	0.041					
Introjected regulation	Constant		12.498	0.000	0.042	0.035	5.899	0.044	0.041
	FGRSS Total	0.204	2.429	0.016					
Intrinsic motivation	Constant		12.996	0.000	0.029	0.022	4.108	0.030	0.029
	FGRSS Total	0.171	2.027	0.045					

*p* < 0.05*.

## 4 Discussion

This study examined the extent to which female athletes’ perceptions of gender role stress influence their sport motivation, with findings evaluated in relation to years of sports participation and the dimensions of the scales employed.

The results revealed that participants’ perceptions of gender role stress were related to their motivation for sports. Accordingly, it can be suggested that the social norms imposed on women shape the psychological burden in sport and highlight the presence of a socially constructed motivational framework. Previous research has also shown that stress perceptions based on gender norms may have important effects on individuals’ psychological stance and motivational levels in sports contexts (32, 55). In this regard, it should be noted that stress is not always a negative parameter but can contribute positively to motivation when experienced at an optimal level (56, 57). In this sense, female athletes experiencing gender role-related stress might interpret this pressure as a challenge rather than a threat, thereby transforming stress into a motivational driver. On the other hand, contrary to the present findings, Bayer, et al. (58) reported that motivation and gender roles do not interact. This discrepancy may be explained by the balancing of gender differences through processes of social modernization. Another possible explanation could be the role of social desirability bias in self-report data, where female athletes may underreport gender role stress to align with socially progressive expectations. Moreover, the interaction between perceived competence and gender identity may buffer the negative effects of gender role stress on motivation.

Regression analyses indicated low-level effects on internalized motivational dimensions such as identified and internal regulation. From the perspective of Self-Determination Theory (59), these results suggest that female athletes derive a sense of autonomy and competence from sport participation, which transforms externally imposed stress into self-determined motivation. Furthermore, in the Turkish sociocultural context, where gender norms remain relatively traditional, participation in sports may function as a subtle form of social resistance and self-empowerment. While this is not explored in the current study, it can contribute theoretically to the interpretation of the study. Similar patterns have been observed in studies from other patriarchal cultures (60, 61), indicating a universal mechanism where sport acts as both a psychological and sociocultural coping strategy for women. The relatively small effect sizes may be related to women’s socio-economic empowerment (60) and the fact that participants’ motivational orientations are shaped more by factors such as coach-team relationships, family, and internal resources than by gender norms (62–66). In this context, sport is widely recognized to exert positive effects in mitigating stress-related problems (67). Therefore, in the current sample, gender norms did not undermine motivation but rather appeared to strengthen athletes’ minimal commitment (61). This may be linked to women’s tendency to adopt a “fight rather than flight” stance in the face of social challenges.

Regarding years of sports experience, no significant relationship was found with gender role stress. Stress is defined as the individual’s response to potential threats from the environment (68, 69). Consistent with this, several studies have indicated that athletic experience does not significantly affect stress levels (70). In sports contexts, stress is directly connected to pressures, perceptions of competition, and performance expectations (71). Beyond athletic performance, however, stress also emerges from socially imposed roles (24), creating additional stressors for athletes (31). Nevertheless, no strong evidence exists in the literature regarding changes in gender role stress as a function of years of participation in sport. This suggests that gender role stressors are more closely related to societal expectations and cultural norms rather than athletic background or experience.

When the relationship between years of sports experience and the sport motivation was examined, no significant correlations were found with amotivation, external regulation, and introjected regulation. This finding does not support the expectation that years of sports experience might be linked to amotivation. Motivation is defined as the driving force that initiates and sustains behavior (72), whereas amotivation reflects feelings of inefficacy and lack of perceived control over behavior (73, 74). In contrast to the current findings, some studies have reported that amotivation decreases as years of participation increase (75, 76). However, other studies consistent with the present research have also found no significant relationship between activity duration and amotivation (77). This discrepancy may be due to the fact that participants in this study were active athletes who voluntarily engaged in sport. Similarly, the absence of significant relationships in external and introjected regulation may reflect participants’ ability to neutralize external pressures. Supporting this, some studies have also reported no significant associations in these dimensions (76).

Conversely, significant relationships were identified with intrinsic motivation, identified regulation, and integrated regulation. These results suggest that motivation is not primarily determined by external factors but is directly linked to intrinsic needs and psychological processes (53, 59, 78). Based on these findings, it may be concluded that motivation types can develop as years of sports participation increase.

## 5 Conclusion

The present study investigated the effects of gender role stress perceptions on female athletes’ sport motivation. The findings demonstrated relationships between gender role stress and the internalized dimensions of motivation. Regression analyses indicated that gender role stress explained a limited proportion of variance in these dimensions. Nevertheless, overall, the results suggest that as female athletes’ perceptions of gender role stress minimally increase, their motivation toward sport also increases. This finding indicates that despite gender-based pressures, female athletes may use sport as a coping strategy, highlighting its supportive role in enhancing motivation. These findings also have practical implications for coaches and sport psychologists working with female athletes in patriarchal societies such as Türkiye. Interventions that emphasize autonomy support and gender-sensitive coaching practices may enhance motivation and resilience, transforming gender role stress into a constructive psychological resource.

### 5.1 Limitations

First, the study was conducted with a limited sample size and a participants (female athletes within a specific age range) from a single university. This limitation limits the generalizability of the findings to different cultures, populations, and age groups. Furthermore, the fact that the participants participated in different sports disciplines is a limitation of the study. While no significant differences were observed in the preliminary analysis, the possibility that humans, as social individuals, can be influenced by their environment persists. Therefore, motivation and stress factors could not be examined in terms of differences in sports disciplines. Because data were collected via self-report scales, participants’ responses may have been biased. Additionally, the cross-sectional design of the study precludes causal inferences between variables. A simple linear regression model was used in the study. This model may be inadequate for testing more complex relationships, such as gender role stress and motivation in sports. In this context, the use of methods such as path analysis may be a limitation of this study.

### 5.2 Recommendations

This study examined only gender role stress and sport motivation dimensions. Future research may broaden the scope by including additional psychosocial factors (e.g., perceived social support, adaptation to cultural norms). Additionally, a study in which branch diversity is limited can be conducted and comparisons can be made with a balanced sample. Employing experimental or mixed-method designs could provide insight into the long-term effects of gender role stress on motivation. Moreover, qualitative approaches may enrich understanding by capturing athletes’ lived experiences.

From a practical perspective, it is recommended that coaches, academics, and sport psychologists:

- Feedback supporting athletes’ autonomy is available.
- Distributing conversations to emphasize gender roles and encouraging them through experience.
- Changing clear referral pathways to psychologists and counselors.
- Differences in brief screening practices to address unbalanced motivation and stress.

## Data Availability

The raw data supporting the conclusions of this article will be made available by the authors, without undue reservation.
